# Supplementary material for: ZNF281 Promotes Colon Fibroblast Activation in TGFβ1-Induced Gut Fibrosis
Source: Int J Mol Sci. 2022 Sep 6;23(18):10261. doi: 10.3390/ijms231810261 (PMC9499662; doi:10.3390/ijms231810261)
Supplement: Supplementary file 1 [file ijms-23-10261-s001.zip › ijms-1878203-Supplementary.pdf]

Supplementary Data

List of murine and human primers

**Species**	**Gene**	**Forward Primer (5'>3')**	**Reverse Primer (5'>3')**
mus musculus	Col3a1	GCCCACGCCTTCTACAC	CCAAGCTCGGTCACACTGACA
	E-cad	CAGCCGGTCTTTGAGGGATT	GGTAACTCTCTCGGTCCAGC
	Fn1	AAGAGGTTGTGACTGTGG GC	ATGGCGTAATGGGAAACCGT
	Il-6	CAAGTCGGAGGCTTAATTACACATG	TGCCATTGCACAACTCTTTCCT
	Mmp9	TGTCTGGAGATTCGACTTGAAGTC	TGAGTTCCAGGGCACACCA
	Rpl32	TGTGCAACAAATCTTACTGTGCT	TGCACACAAGCCATCTACTCA
	Snail	GCGGAGTTGACTACCGACC	GAAGGTGAACTCCACACACG
	Tgfβ1	TGGCGATACCTCAGCAACC	CTCGTGGATCCACTTCCAG
	Tnf-α	CAGACCCTCACACTCCAGATCATCTT	CCACTTGGTGGTTTGCTACGA
	Vim	AGACCAGAGATGGACAGGTGA	TTGCGCTCCTGAAAAACTGC
	αSma	ACCATTGGAAACGAACGCT	TTTCGTGGATGCCCGCTG
homo sapiens	ADAM19	GCAATGCCTCTAATTGTACCCTG	GAGCCAACAGCTTACACTGG
	COL1A1	TGATGGGATTCCCTGGACCT	TCCAGCCTCTCCATCTTTGC
	COL3A1	TCGAGGCAGTGATGGTCAAC	GGTCCAACTTCACCCTTAGCA
	COL4A1	GGTGTTGCAGGAGTGCCAG	GCAAGTCGAAATAAAACTCACCAG
	CTGF	AGGAGTGGGTGTGTGACGA	CCAGGCAGTTGGCTCTAATC
	FAP	CCCACGCTCTGAAGACAGAA	AGTTATGAACTCTTGAAGGGCGT
	FN1	AGACCATACCTGCCGAATGTAG	GAGAGCTTCCTGTCCTGTAGAG
	GAPDH	GAAATCCCATCACCATCTTCCAGG	GAGCCCCAGCCTTCTCCATG
	ITGB3	CATGGATTCCAGCAATGTCCTCC	TTGAGGCAGGTGGCATTGAAGG
	SERPINE1	CACAAATCAGACGGCAGCAC	GGGCGTGGTGAACTCAGTATAG
	SNAIL	CTCTAGGCCCTGGCTGCTA	AGTGGGGACAGGAGAAGGG
	THBS1	GCTGGAAATGTGGTGCTTGTCC	CTCCATTGTGGTTGAAGCAGGC
	ZNF281	GCCATCCTCTCCCCAAGTC	GAGCTTCGGAAAGCAGCACTA
	αSMA	CCGACCGAATGCAGAAGGA	ACAGAGTATTTGCGCTCCGAA

Supplementary Figures and Tables

Supplementary Figures

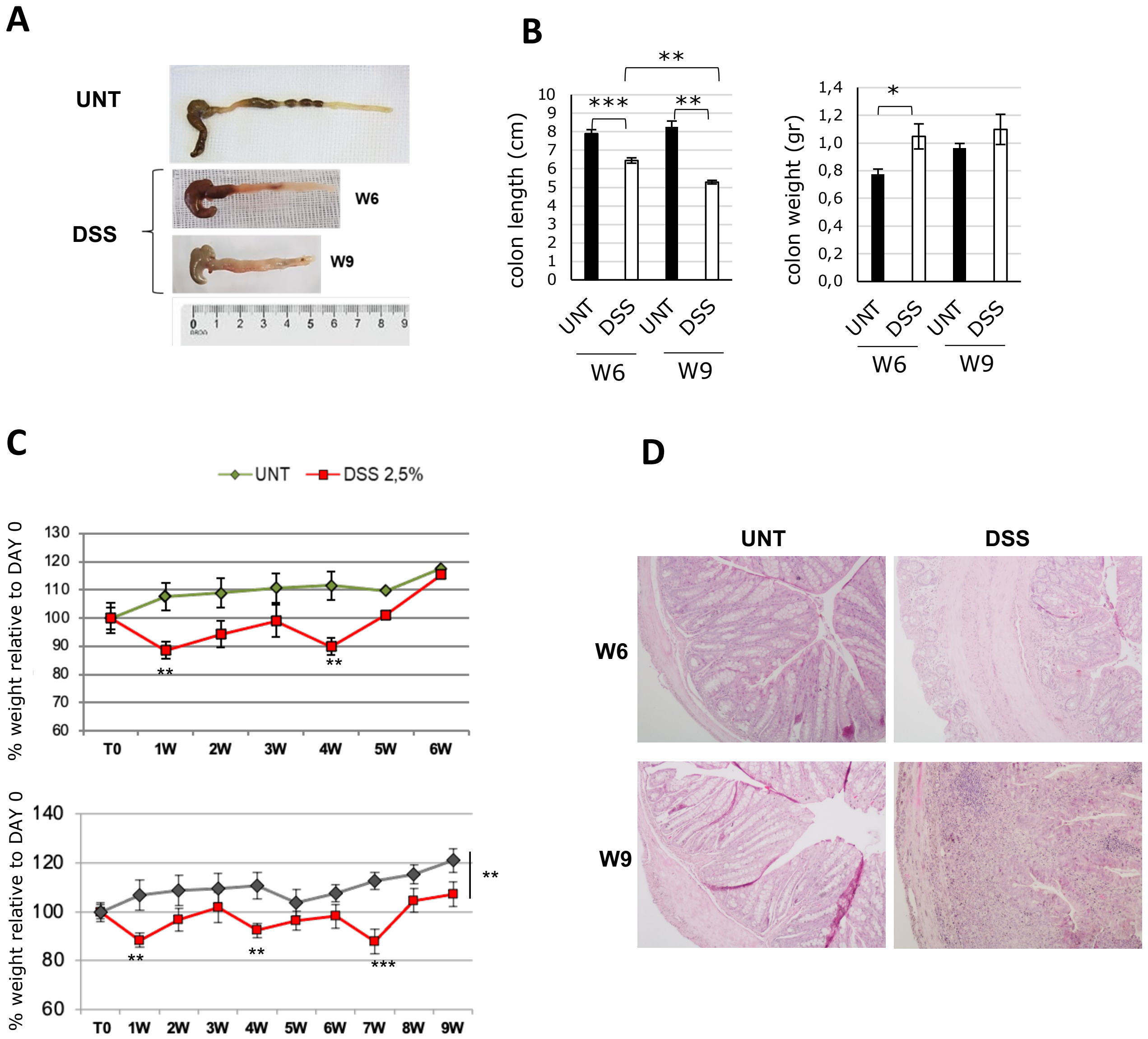

Figure S1. C57BL/6J male mice were subjected to cycles of 2.5% DSS (dextran sulfate sodium) for 2 weeks followed by 1 week of recuperation to establish mice intestinal fibrosis model. DSS-treated mice were sacrificed at 6 (DSS W6; n=8) and 9 weeks (DSS W9; n=5) of treatment along with untreated controls (UNT; n=8). To verify the progression of chronic intestinal inflammation and fibrosis, colon length and weight (A and B), body weight (C) were measured. Hematoxylin and Eosin staining (D) confirmed that DSS successfully induced chronic intestinal fibrosis. Data are expressed as mean ± SEM. *= p-value≤ 0.05; **= p-value≤ 0.01; ***= p-value≤ 0.001

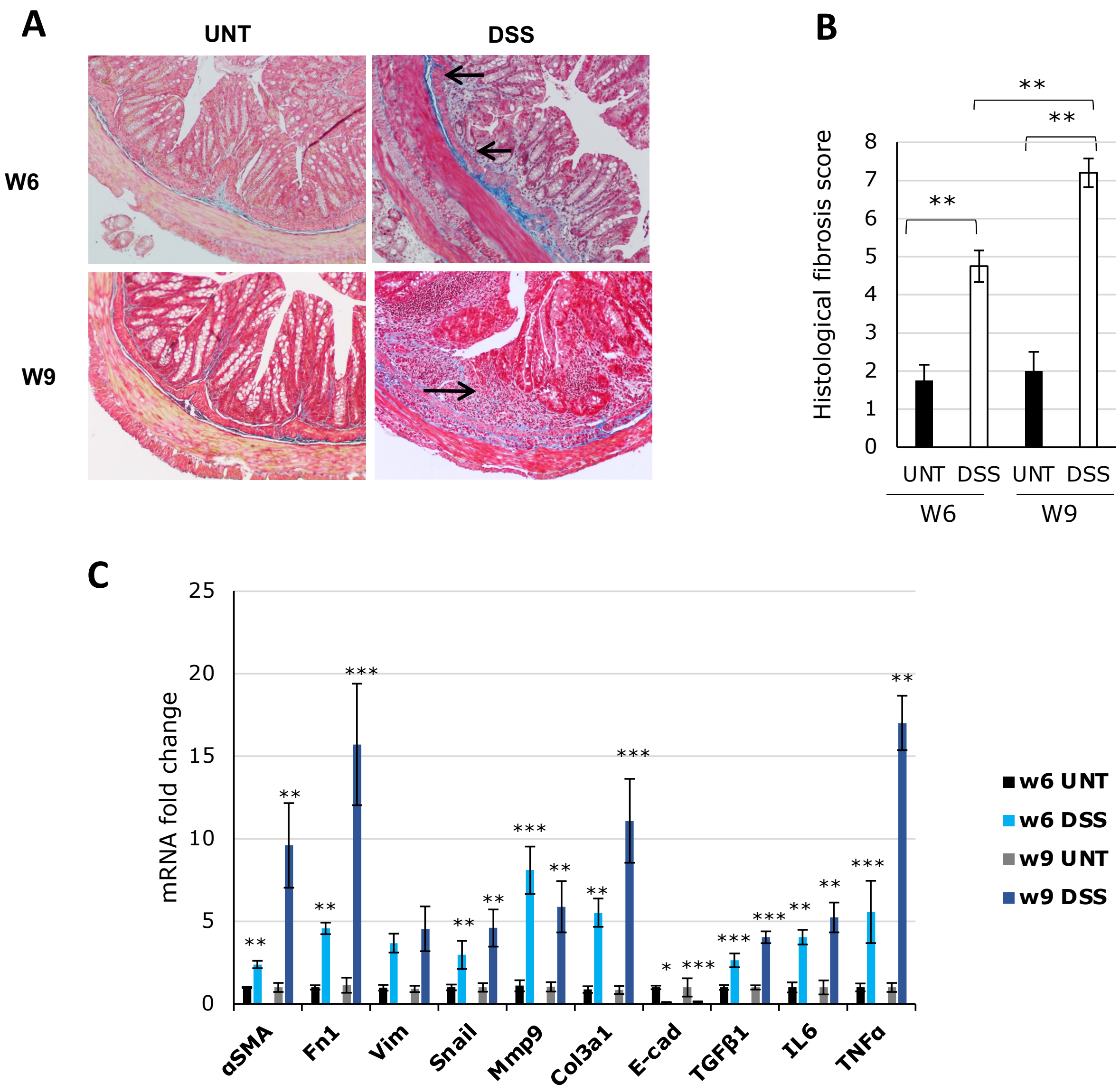

Figure S2. Masson's trichrome stain was assessed on murine colon sections (A, *black arrows* indicate collagen fiber deposition) and histological fibrosis scoring was calculated (B). mRNA expression analysis of fibrosis and inflammation markers by qRT-PCR confirmed occurrence of intestinal fibrosis. UNT, untreated animals; DSS, dextran sulfate sodium; W6, week 6 of treatment; W9, week 9 of treatment. Data are expressed as mean ± SEM. *= p-value≤ 0.05; **= p-value≤ 0.01; ***= p-value≤ 0.001

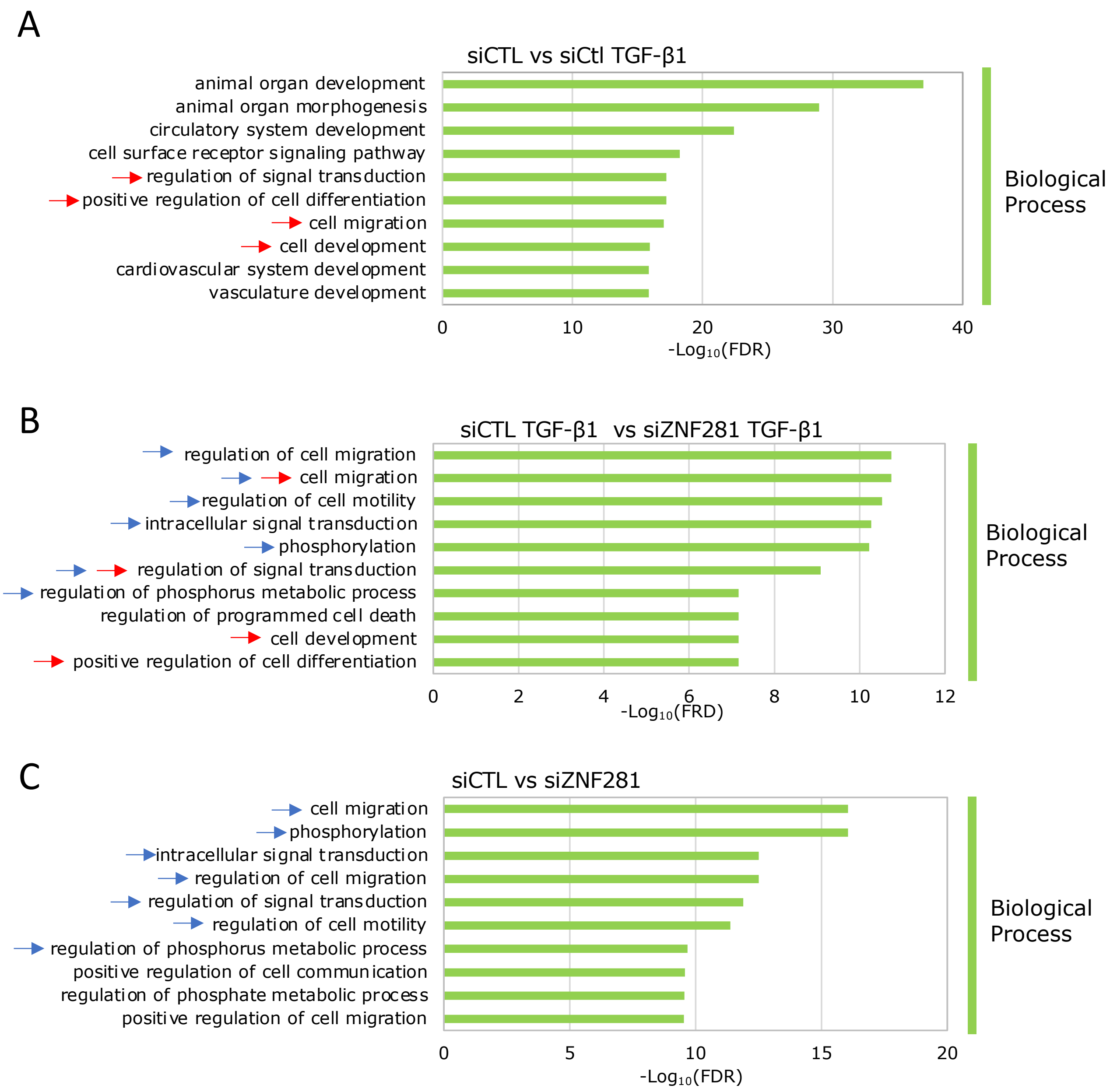

Figure S3. Top-10 Gene Ontology (GO) terms enriched in DEGs between siCtl and siCtl TGFβ1 (A), siCtl TGFβ1and siZNF281 TGFβ1 (B) and siCtl and siZNF281 (C) treated cells in the Biological Process (BP) category. *Red arrows* indicated GO terms shared between A and B; *blue arrows* indicated GO terms shared between B and C.

A

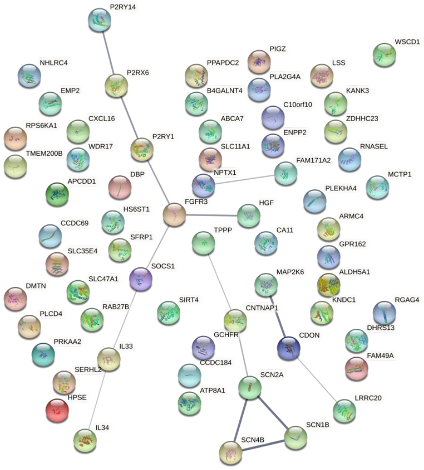

B

Pathway name	Entities			
	found	ratio	p-value	FDR*
Interaction between L1 and Ankyrins	4 / 33	0.002	3.65e-05	0.006
Phase 0 - rapid depolarisation	4 / 34	0.002	4.09e-05	0.006
L1CAM interactions	6 / 130	0.009	8.71e-05	0.008
FGFR3 mutant receptor activation	3 / 17	0.001	1.22e-04	0.008
Signaling by activated point mutants of FGFR3	3 / 17	0.001	1.22e-04	0.008

Figure S4. Functional analysis of 71 genes from Group 2m, downregulated by TGFβ1via ZNF281. Protein-protein interaction (PPI) network was built by STRING (https://string-db.org).The edges indicate both functional and physical protein associations (A). Biological pathway enrichment was performed using Reactome (https://reactome.org) (B).

Supplementary Tables

Table S1. Differentially expressed genes (DEGs) between CCD18-Co cells exposed to TGFβ1 (siCtl TGF β1) or not (siCtl), after ZNF281 silencing (siZNF281; siZNF281 TGFβ1), as assessed by RNA-sequencing (adj P-value < 0.05, log2FC >1.0)

Table S2. Gene Ontology (GO) term enrichment analysis of DEGs between siCtl and siCtl TGFβ1 treated cells (FDR< 0.05).

Table S3. GO term enrichment analysis of DEGs between siCtl TGFβ1 and siZNF281 TGFβ1 treated cells (FDR< 0.05).

Table S4. GO term enrichment analysis of DEGs between siCtl and siZNF281 treated cells (FDR< 0.05).

Table S5. GO term enrichment analysis of DEGs belonging to Group 2 (FDR< 0.05).

Table S6. GO term enrichment analysis of DEGs belonging to Group 1 and 3 (FDR< 0.05)

Table S7. Genes belonging to Group 2 including genes on which ZNF281 silencing and TGFbeta1 treatment have opposite effects. ZNF_log2_FC, log2 fold-change in siCtl vs siZNF281; ZNF_Adj_p-value, adjusted p-value in siCtl vs siZNF281; TGF_log2_FC log2 fold-change in siCtl vs siCtl TGFβ1; TGF_Adj_p-value, adjusted p-value in siCtl vs siCtl TGFβ1.

Table S8. GO term enrichment analysis (FDR< 0.05) of 102 genes up-regulated by TGFβ1 (siCtl vs siCtl TGFβ1) and downregulated when ZNF281 was silenced (siCtl vs siZNF28) (adj P-value < 0.05, log2FC >1.0).
